# Time of Day Matters: An Exploratory Assessment of Chronotype in a Forensic Psychiatric Hospital

**DOI:** 10.3389/fpsyt.2020.550597

**Published:** 2020-12-18

**Authors:** Kimberly D. Belfry, Scott H. Deibel, Nathan J. Kolla

**Affiliations:** ^1^Waypoint Centre for Mental Health Care, Waypoint Research Institute, Penetanguishene, ON, Canada; ^2^Department of Psychology, Memorial University of Newfoundland, St. John's, NL, Canada; ^3^Department of Psychiatry, University of Toronto, Toronto, ON, Canada; ^4^Centre for Addiction and Mental Health (CAMH), Toronto, ON, Canada; ^5^Violence Prevention Neurobiological Research Unit, CAMH, Toronto, ON, Canada

**Keywords:** chronotype, morningness–eveningness, evening type, forensic, psychotic disorders, aggression, substance use disorder, dark triad

## Abstract

A growing body of evidence links the late chronotype to mental illness, aggression, and aversive personality traits. However, much of what we know about these associations is based on healthy cohorts, and it is unclear how individuals with high levels of aggression, including forensic psychiatric populations, but not offenders, are affected. The present study aimed to measure chronotype in a forensic psychiatric inpatient population, evaluate the impact of diagnosis, and identify any interactive relationships between chronotype, diagnosis, aggression, and dark triad traits. Subjects completed the reduced Morningness–Eveningness Questionnaire (rMEQ), Munich ChronoType Questionnaire (MCTQ), Pittsburgh Sleep Quality Index (PSQI), Buss Perry Aggression Questionnaire–Short Form (BPAQ-SF), and Short Dark Triad Questionnaire (SD3). We sampled 55 forensic psychiatric patients (52 males) between the ages of 23 and 73 years (mean ± SD: 39.6 ± 14.3 years). Among the patients sampled, 25% were evening types and 36% were morning types. Eveningness was greater in patients with a personality disorder; however, no chronotype differences were found for psychosis patients. Patients without psychosis had a positive association between anger and eveningness, as well as between hostility and eveningness. For subjects with a substance use disorder, morningness was positively associated with narcissism. Conversely, an association between eveningness and greater narcissism was identified in patients who did not have a substance use disorder. These findings suggest that, compared to the general population, evening types are more prevalent in forensic psychiatric populations, with the strongest preference among patients diagnosed with a personality disorder. No differences in chronotype were identified for psychosis patients, which may be related to anti-psychotic medication dosing. Given the sex distribution of the sample, these findings may be more relevant to male populations.

## Introduction

Sunrise and sunset are some of the most salient environmental cues that influence an organism's behavior (1). Circadian rhythms allow organisms to adjust their physiology and behavior so that they are synchronized with the environment (1). Although humans are very active during the day and less so during the night, there is variation in when one prefers to go to bed, wake up, and perform cognitive and physical activities. Chronotype refers to the timing of these preferences in relation to sunrise and sunset (2). Early chronotypes are early to rise and sleep, with a preference for cognitive and physical activities in the morning. Conversely, late chronotypes are late to rise and sleep, with a preference for cognitive and physical activities later in the day.

While the relationship between mental illness and the late chronotype has been reported in some studies (3, 4), findings in individuals with psychosis are equivocal (5). One study reported an increased proportion of eveningness in schizophrenia outpatients, relative to healthy controls (6), whereas others have found a positive correlation between eveningness and young persons with a psychotic disorder (7). Conversely, chronotypes among individuals with psychosis have also been observed as being similar to or having greater morning preference, relative to healthy controls (8, 9). Whether psychosis and chronotype relate to aggression remains unknown.

The late chronotype has been implicated as a transdiagnostic correlate of substance use and maladaptive behaviors such as aggression (3). One such study looked at chronotype among substance use disorder in outpatients and found that eveningness was significantly associated with addiction, but not with addiction severity and that morningness was associated with patients comorbid for antisocial personality disorder (10). Conversely, two reviews of the chronotype literature associated eveningness with antisocial and aggressive behaviors in children and adolescents (11) as well as eveningness with psychopathologies in young-adult and adult populations (12). Likewise, a study of the general adult population found that evening types were more impulsive and experienced more anger, whereby the role of impulsivity potentially influenced the extent to which chronotype predicted anger (13).

Socially aversive traits, such as Machiavellianism, narcissism, and psychopathy, otherwise known as the dark triad traits, have shown a relationship with eveningness (14, 15). Machiavellianism is characterized by manipulation and exploitation of others and a general lack of morality; narcissism is associated with beliefs of grandiosity, self-entitlement, dominance, and superiority; and psychopathy is exemplified by a lack of empathy, combined with high impulsivity or risk-prone behaviors (16, 17). Clinically significant levels of narcissism and psychopathy may be diagnosed as narcissistic personality disorder and antisocial personality disorder (ASPD), respectively (18). While Machiavellianism is not a personality disorder, the dark triad has been uniquely associated with personality disorders, as determined by the Personality Inventory for the DSM-5 (19), suggesting distinct profiles for each dark triad trait in regard to clinically maladaptive traits (20). Dark triad traits also play a pivotal role in offending patterns, as individuals higher on the dark personality spectrum are deceitful, amoral, with shallow empathy and reduced guilt after moral transgressions, and thus are more likely to bend social rules and, in some cases, perpetrate crimes (21, 22). To the best of our knowledge, no research to date has examined the relationship between chronotype and the dark triad in forensic psychiatric patients. This is one of the unique aspects of the current study. However, one study of university students found that men scored higher on dark triad traits than women and that chronotype significantly mediated the effect of sex on dark triad scores, particularly for Machiavellianism and psychopathy (14).

Very little is known about chronotypes and mental illness in forensic psychiatric populations. The higher prevalence of aggression, greater severity of mental illness, and longer duration of institutionalization represent a unique opportunity to evaluate chronotype in forensic psychiatric populations (23, 24). Much of the existing body of research has focused on adolescent and college-aged populations, groups that are less likely to exhibit high rates of aggression. Studies that have investigated chronotypes in inpatient populations have been mostly limited to depression (25, 26). Therefore, the aims of the current study were the following: (i) to provide a novel assessment of chronotype in a forensic psychiatric patient population; (ii) to compare chronotype across a variety of diagnoses; and (iii) to investigate the interactive relationships between chronotype, diagnosis, aggression, and dark triad traits.

## Methods

### Setting

Waypoint Centre for Mental Health Care (Waypoint) is a tertiary care psychiatric hospital with 180 beds allocated for forensic psychiatric inpatients. There are 160 beds for male inpatients under high-security, and 20 beds for male and female patients under medium-security.

### Participants

We aimed to sample the entire forensic population, but required that subjects be able to provide informed consent to be included. Therefore, the presence of acute psychosis, major cognitive impairment, or significant developmental delay, was considered exclusion criteria. To recruit study participants, information sessions were provided and advertisements were posted in common areas of the hospital. Of the 80 patients who were approached, 58 forensic inpatients were successfully recruited to participate in the present study, three of whom lacked the intellectual capacity to complete the questionnaires and were thus excluded from the analysis (see Table 1 for sample characteristics). Patients who chose not to participate cited unwillingness to disclose personal information or a lack of motivation to complete the questionnaires. All participants provided written consent before participating in the study.

**Table 1:** Demographic characteristics, BPAQ-SF, and SD3 mean scores.

		***n* = 55**
Age (years), *M (SD)*		39.6 (14.3)
Sex, % (*n*)	Male	94.5% (52)
	Female	5.5% (3)
Ethnicity, % (*n*)	White	50.9% (28)
	Black	16.4% (9)
	Asian	7.3% (4)
	First Nations	3.6% (2)
	Middle Eastern	3.6% (2)
	Mixed Heritage	3.6% (2)
	Latin American	1.8% (1)
	Not provided	12.7% (7)
Security level, % (*n*)	Medium	12.7% (7)
	High	87.3% (48)
Anti-psychotic dose (CPZ equiv.), *M (SD)*		600 (525)
Diagnosed disorder, % (*n*)	Psychotic	76.8% (43)
	Anxiety	7.1% (4)
	Personality	39.3% (22)
	Substance use	53.6% (30)
	Neurodevelopmental	19.6% (11)
	Sexual	10.7% (6)
	Other	1.8% (1)
Index offense, % (*n*)	Murder or attempted murder	28.6% (16)
	Physical assault	51.8% (29)
	Verbal assault	32.1% (18)
	Sexual offenses	14.3% (8)
	Reckless endangerment	10.7% (6)
Length of stay (years), *M (SD)*		5.3 (9.5)
BPAQ-SF, *M (SD)*	Physical aggression	6.2 (3.1)
	Verbal aggression	7.1 (3.7)
	Anger	6.7 (3.4)
	Hostility	8.1 (3.8)
SD3, *M (SD)*	Machiavellianism	3.0 (0.6)
	Narcissism	3.1 (0.6)
	Psychopathy	2.5 (0.6)

*CPZ equiv., daily scheduled intake of anti-psychotic medication in chlorpromazine equivalents; Cumulative diagnosed disorder and index offense are greater than 100% due to the presence of comorbidity and multiple offenses per participant; BPAQ-SF, Buss Perry Aggression Questionnaire—Short Form; SD3, Short Dark Triad Questionnaire*.

The daily and weekly routines of all the participants are structured according to the therapy provided by the hospital. Medication is administered at 7:30, 11:30, 16:30, and 20:30; meal times are at 7:45, 11:45, and 16:45; and snacks are provided at 10:30, 14:30, and 20:30. Patients are able to shower between the hours of 8:30 and 10:00 on an every other day basis, following which hospital programming is offered between the hours of 10:00 and 16:00 on weekdays. Examples of the programs include stretch group, narcotics anonymous, emotional regulation coaching, or scheduled physical activity in the gymnasium or pool facilities at the hospital. Patients also have the option to go outside for at least 1 h per day. Patients have daily access to a physician or nurse practitioner and appointments for other services, including psychotherapy, chiropody, dentistry, and hairdressing, which are scheduled on an as-needed basis. Some patients are paid for vocational work, where shifts are a maximum of 3 h per day. All patients are required to be in their rooms by 22:00, but are free to read or otherwise occupy themselves prior to going to sleep. On Saturday and Sunday, meal service and medication times remain the same, but no other activities are typically scheduled as allied staff are not present on the hospital premises on weekends.

### Ethics

The Waypoint Research Ethics Board approved all components of this study.

### Clinical Data and Self Report

#### Demographic Variables

Participants agreed to the collection of their recorded medical and demographic data, including age, sex, ethnicity, diagnoses, medication at the time of assessment, and index offense (Table 1). Most of this information was extracted from the participants' electronic health record (EHR).

#### Diagnoses

Mental health diagnoses were obtained from the EHR and were based on information provided by the treating psychiatrist. Patients were grouped into the following categories: psychotic disorders, personality disorders, anxiety disorders, substance use disorders, neurodevelopmental disorders, sexual disorders, and other diagnoses. Categories were not mutually exclusive. That is, patients could belong to multiple categories depending on their comorbidities.

#### Index Offenses

Index offense describes the offending behavior that resulted in the patients' hospitalization. Index offenses were categorized as belonging to the following categories: murder or attempted murder, physical assault, verbal assault, sexual offenses, or reckless endangerment (e.g., arson or dangerous operation of a motor vehicle).

#### Medications

Antipsychotic medications for each participant were converted to chlorpromazine equivalents for the analysis. Other medications that participants were taking were also recorded.

#### Study Measures

##### Reduced Morningness–Eveningness Questionnaire (rMEQ)

Study participants completed the Horne-Östberg reduced Morningness–Eveningness Questionnaire (rMEQ) (27, 28) to assess chronotype. The rMEQ is a 5-item survey with Likert-type responses that assesses times that one prefers to wake up and go to bed. To measure these questions, participants are asked, “What time would you get up if you were entirely free to plan your day?”; “During the first half-hour after you wake up in the morning, how tired do you feel?”; “At what time in the day do you feel you become tired as the result of need for sleep?”; and “At what time of the day do you think that you reach your ‘feeling best’ peak?”; and “One hears about ‘morning’ and ‘evening’ types of people. Which ONE of these types do you consider yourself to be?” Scores range from 4 to 25, where lower numbers are associated with a stronger preference for eveningness, and higher numbers are indicative of morningness. rMEQ scores ranging from 4 to 7 were classified as “definite evening type”; 8 to 11 as “moderate evening type”; 12 to 17 as “intermediate type”; 18 to 21 as “moderate morning type”; and 22 to 25 as “definite morning type” chronotypes (28). The original MEQ similarly assesses morningness–eveningness but includes additional items that evaluate when one prefers to engage in physical and mental activities, as well as sleep preferences that would result from staying awake outside of one's normal routine. The rMEQ has comparable sensitivity and can be used to reliably classify chronotype (28).

##### Munich ChronoType Questionnaire (MCTQ)

The Munich ChronoType Questionnaire (MCTQ) (29) is a self-report questionnaire that evaluates chronotype by comparing sleep times on work and free days. In the context of this study, “work days” were considered weekdays, when the daily schedule was determined according to therapeutic activities provided by the hospital; and “free days” were the relatively less structured weekend days. Unlike the rMEQ that measures chronotype according to preference, the MCTQ measures chronotype according to sleep behavior. Specific measurements include sleep mid-point on work days (MSW), sleep mid-point on free days (MSF), sleep mid-point adjusted for work day sleep debt (MSF_SC_), and social jetlag (difference between MSW and MSF). Adjusted sleep midpoint (MSF_SC_) approximates chronotype by considering sleeplessness during the week, while social jetlag informs about the relative impact that free-day sleep behavior has on workdays. The MCTQ is designed to assess chronotype according to typical sleep times 1 month prior to the survey. For the purposes of this study, we chose to exclude participant responses if their hospitalization was <14 days (*n* = 6); this approach has been previously published (30).

##### Pittsburgh Sleep Quality Index (PSQI)

To assess sleep quality, patients were asked to complete the Pittsburgh Sleep Quality Index (PSQI) (31). The PSQI assesses sleep quality according to disturbances over a 1-month period. Using information provided by the participant, the PSQI generates component scores for subjective sleep quality, sleep latency, sleep duration, habitual sleep efficiency, sleep disturbances, use of sleeping medication, and daytime dysfunction. Global scores of 5 or higher are reflective of “poor sleep.” PSQI uses the preceding 4 weeks to evaluate one's sleep quality. Similar to MCTQ, we excluded PSQI responses if participant stay was <14 days (*n* = 6) (32).

##### Buss Perry Aggression Questionnaire-Short Form (BPAQ-SF)

Aggression was measured using the Short-Form Buss Perry Aggression Questionnaire (BPAQ-SF) (33, 34). The BPAQ-SF is a self-report, 12-question-assessment that uses Likert-type questions to determine scores for physical aggression, verbal aggression, anger, and hostility. Participants are asked to individually rate, from 1 to 5, how characteristic each item statement is of them (“extremely uncharacteristic of me” = 1; “extremely characteristic of me” = 5). Equal weight is given to each sub-trait, where physical aggression, verbal aggression, anger, and hostility items include, “Given enough provocation, I may hit another person,” “I often find myself disagreeing with people,” “I have trouble controlling my temper,” and “Other people always seem to get the breaks,” respectively. The original BPAQ uses 29 Likert-type questions, which uses nine items specific to physical aggression, five for verbal aggression, seven for anger, and eight for hostility. The shortened version was created to eliminate items that were relatively unreliable indicators of the dimensions that they were intended to reflect, while maintaining the conceptual definitions of the original factors. The BPAQ-SF has been validated in undergraduate populations and is reported to be psychometrically superior to the original scale, according to goodness-of-fit (33).

##### Short Dark Triad Questionnaire (SD3)

Lastly, to assess aversive personality traits, study participants were asked to complete the Short Dark Triad questionnaire (SD3) (35). The SD3 consists of 27 Likert-type questions that measure dark triad personality traits (Machiavellianism, narcissism, and psychopathy).

### Statistical Analysis

Prior to analysis, normality of the data set was visually confirmed using P–P plots. Where data were found to be non-normal, non-parametric analyses were used. Cronbach's alpha was used to assess the internal consistency of the revised questionnaires rMEQ and BPAQ-SF. Chronotype was determined for each study participant using rMEQ cumulative scores. Each subject was categorized as being either definite evening, moderate evening, intermediate, moderate morning, or definite morning. MSW, MSF, MSF_SC_, and social jetlag were computed for each respondent. Participants whose sleep pattern was highly erratic, in terms of sustained periods of wake or sleep (>22 h per day), were considered to be extreme outliers and were, therefore, removed from the analysis (*n* = 2). Linear relationships between rMEQ score and MCTQ sleep mid-points (MSW, MSF, and MSF_SC_), as well as PSQI global scores were investigated using Pearson correlation coefficients. Using chronotype as the dependent variable, analysis of variance (ANOVA) tests were performed to detect group differences between chronotype across demographic variables such as age, ethnicity, and anti-psychotic dose. Due to the high prevalence of comorbid conditions, differences related to social jetlag, MSF_SC_, bed time, nightly hours of sleep time spent in bed, anti-psychotic dose, and rMEQ scores were assessed individually for psychosis, personality disorder, and substance use disorder diagnostic groups using *t*-tests.

ANOVA tests were run using chronotype as the independent variable to look at its influence on aggression and dark triad traits. Correlational analyses were used to detect linear relationships between MSF_SC_, social jetlag, and PSQI global variables, and aggression and dark triad traits. To visualize trends across diagnostic group, scatter plots of rMEQ scores vs. aggression scores (verbal aggression, physical aggression, anger, or hostility) and rMEQ scores vs. dark triad trait scores (Machiavellianism, narcissism, or psychopathy) were created. All data were analyzed using SPSS version 25 (SPSS, Inc., Chicago, IL, USA), where a *p-*value of 0.05 or less was used to determine statistical significance.

## Results

### Reliability of Reduced Questionnaires

Cronbach's alpha scores for reduced measures rMEQ (5 items) and BPAQ-SF (12 items) were 0.70 and 0.88, respectively, indicating adequate internal reliability for the present sample.

### Characteristics of Study Participants

Of the 43 subjects diagnosed with a psychotic disorder, 13 had a comorbid personality disorder, 11 of which also had a substance use disorder. Demographic characteristics, BPAQ-SF, and SD3 mean scores are summarized in Table 1. Using scores obtained from rMEQ responses, participants were classified according to chronotype: definite evening (*n* = 1, 1.8%), moderate evening (*n* = 13, 23.6%), intermediate (*n* = 21, 38.2%), moderate morning (*n* = 11, 20.0%), and definite morning (*n* = 9, 16.4%; Table 2). The demographic/clinical variables age (*p* = 0.917), ethnicity (*p* = 0.995), and anti-psychotic dose (*p* = 0.845) were not associated with chronotype as per ANOVA testing.

**Table 2:** Descriptive statistics according to chronotype and Pearson correlations between rMEQ score and sleep mid-point (MCTQ) and sleep quality (PSQI) scores.

	**Definite evening**	**Moderate evening**	**Intermediate**	**Moderate morning**	**Definite morning**	**rMEQ score**	
	***n*** **=** **1**	***n*** **=** **13**	***n*** **=** **21**	***n*** **=** **11**	***n*** **=** **9**	***r***	***p***
MSW	NA	4:05 (0:54)	3:29 (0:58)	3:00 (0:53)	2:24 (1:20)	−0.49	<0.001
MSF	NA	4:26 (1:08)	4:03 (1:25)	3:33 (0:56)	2:47 (1:32)	−0.39	0.006
MSF_SC_	NA	4:25 (1:07)	3:50 (1:19)	3:28 (0:54)	2:32 (1:32)	−0.43	0.002
PSQI Global	NA	9.8 (3.8)	7.6 (4.4)	6.9 (4.3)	4.4 (1.8)	−0.39	0.007

*rMEQ, reduced Morningness–Eveningness Questionnaire; MCTQ, Munich ChronoType Questionnaire; MSW, Mid-sleep on work days; MSF, Mid-sleep on free days; MSF_SC_, Mid-sleep on free days corrected for sleep debt on work days; PSQI, Pittsburgh Sleep Quality Index; NA, Not available, study participant was an extreme outlier and thus MCTQ and PSQI data were excluded from the analysis*.

### Chronotype, Sleep Mid-points, and Subjective Sleep Quality

Lower rMEQ scores (e.g., greater eveningness) correlated with a later sleep mid-point on working days (MSW; *r* = −0.49, *p* < 0.001), free days (MSF; *r* = −0.39, *p* = 0.006), and free days corrected for sleep debt (MSF_SC_; *r* = −0.43, *p* = 0.002). Greater eveningness was also correlated with greater PSQI global scores (*r* = 0.39, *p* = 0.007), indicating a positive association between eveningness and self-reported poor quality of sleep. PSQI global scores indicated that average sleep quality for moderate evening, intermediate, and moderate morning types was poor (PSQI > 5).

### Diagnostic Group, Sleep, and Chronotype

No differences were identified between the personality disorder and non-personality disorder samples for rMEQ scores (cumulative score ranging from 4–25) (*p* = 0.133), MSF_SC_ (*p* = 0.454), or social jetlag (*p* = 0.595). However, personality disorder chronotypes (definite evening, moderate evening, intermediate, moderate morning, or definite morning) had an increased proportion of evening types (*p* = 0.047; Table 3). Patients with personality disorders in the sample spent less time in bed (*p* = 0.040), slept less hours per night (*p* = 0.015), and were prescribed lower doses of anti-psychotic medication (*p* = 0.019), when compared to the non-personality disorder population (Table 3).

**Table 3:** Differences in chronotype, anti-psychotic medication, hours slept, hours spent in bed, and rMEQ scores for personality disorder and no personality disorder groups.

	**Personality disorder**	**No personality disorder**	
	***n=*** **22**	***n=*** **33**	***p***
			**ANOVA**
Definite morning	2	7	0.047
Moderate morning	4	7	
Intermediate	7	14	
Moderate evening	8	5	
Definite evening	1	0	
			*t-*Test
rMEQ score, *M (SD)*	14.3 (5.4)	16.4 (4.8)	0.133
MSF_SC_ (time), *M (SD)*	3:52 (1:13)	3:34 (1:26)	0.454
Social jetlag (minutes), *M (SD)*	32.3 (52.9)	25.8 (48.0)	0.595
Bed time, *M (SD)*	22:33 (1:24)	21:50 (1:15)	0.081
Hours in bed, *M (SD)*	9.0 (2.1)	10.2 (1.8)	0.040
Hours slept, *M (SD)*	7.2 (2.5)	8.7 (1.6)	0.015
Anti-psychotic dose (CPZ equiv.), *M (SD)*	399 (422)	743 (568)	0.019

*rMEQ, reduced Morningness–Eveningness Questionnaire; low rMEQ scores indicate preference eveningness and high scores indicate preference for morningness; ANOVA, analysis of variance; MSF_SC_, Mid-sleep on free days corrected for sleep debt on work days; CPZ equiv., daily scheduled intake of anti-psychotic medication in chlorpromazine equivalents*.

Patients with a psychotic diagnosis did not differ from the group without a psychotic disorder in terms of rMEQ raw scores (*p* = 0.731), distribution of chronotypes (*p* = 0.773), MSF_SC_ (*p* = 0.120), or social jetlag (*p* = 0.190; Table 4). Psychosis patients spent more time in bed (*p* = 0.001), slept longer (*p* < 0.001), and had a higher mean anti-psychotic dose (*p* < 0.001; Table 4).

**Table 4:** Differences in chronotype, anti-psychotic medication, hours slept, hours spent in bed, and rMEQ scores for psychosis and non-psychosis groups.

	**Psychosis**	**Non-psychosis**	
	***n=*** **43**	***n=*** **12**	***p***
			**ANOVA**
Definite morning	6	3	0.773
Moderate morning	8	3	
Intermediate	19	2	
Moderate evening	10	3	
Definite evening	0	1	
			*t-*Test
rMEQ score, *M (SD)*	15.4 (4.7)	16.0 (6.6)	0.731
MSF_SC_ (time), *M (SD)*	3:48 (1:21)	2:56 (1:10)	0.120
Social jetlag (minutes), *M (SD)*	5.7 (15.1)	32.2 (52.4)	0.190
Bed time, *M (SD)*	22:00 (1:08)	22:42 (2:11)	0.440
Hours in bed, *M (SD)*	10.1 (1.7)	7.6 (2.4)	0.001
Hours slept, *M (SD)*	8.6 (1.6)	5.4 (2.3)	<0.001
Anti-psychotic dose (CPZ equiv.), *M (SD)*	736 (520)	138 (289)	<0.001

*rMEQ, reduced Morningness–Eveningness Questionnaire; low rMEQ scores indicate preference eveningness and high scores indicate preference for morningness; ANOVA, analysis of variance; MSF_SC_, Mid-sleep on free days corrected for sleep debt on work days; CPZ equiv., daily scheduled intake of anti-psychotic medication in chlorpromazine equivalents*.

Individuals diagnosed with a substance use disorder did not differ from the non-substance use disorder population in terms of anti-psychotic medications, sleep, or chronotype (*p* > 0.05).

### Relationship Trends Between Chronotype and Aggression and Dark Triad Traits

ANOVA tests determined that chronotype was not associated with aggression (*p-*value range 0.102–0.734), but a minor effect was identified for dark triad traits (Machiavellianism, *p* = 0.045; narcissism, *p* = 0.962; psychopathy, *p* = 0.214). In the case of Machiavellianism, intermediate chronotypes were most likely to have the highest scores. Pearson correlations determined no significant relationship between MSF_SC_ and aggression (*p*-value range 0.495 to 0.951) or dark triad traits (*p*-value range 0.186 to 0.456); PSQI global score was not related to aggression (*p*-value range 0.058 to 0.700) or dark triad traits (*p*-value range 0.326 to 0.523). Spearman rank correlations identified no significant linear relationships between social jetlag and aggression (*p-*value range 0.346 to 0.965); and social jetlag and dark triad traits (*p*-value range 0.396 to 0.606).

**Figure 1:**
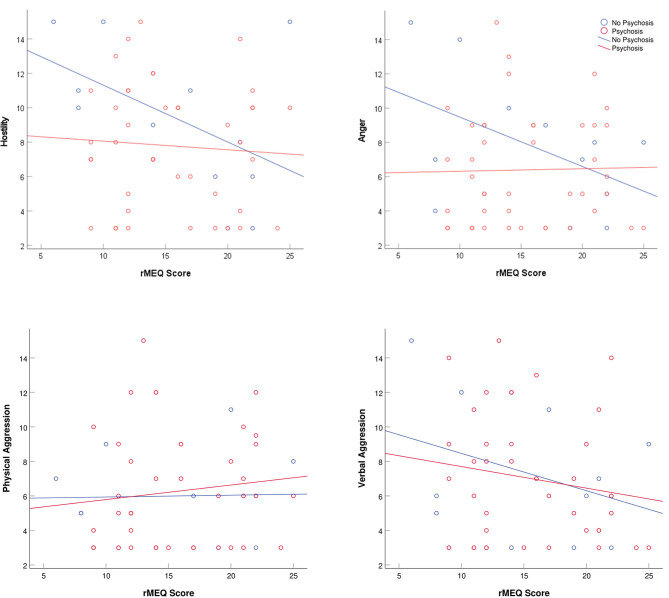
Scatter plots of Buss Perry Aggression Questionnaire-Short Form (BPAQ-SF) subscores and reduced Morningness–Eveningness Questionnaire (rMEQ) scores separated by psychosis and non-psychosis populations. Low rMEQ scores indicate preference eveningness and high scores indicate preference for morningness.

**Figure 2:**
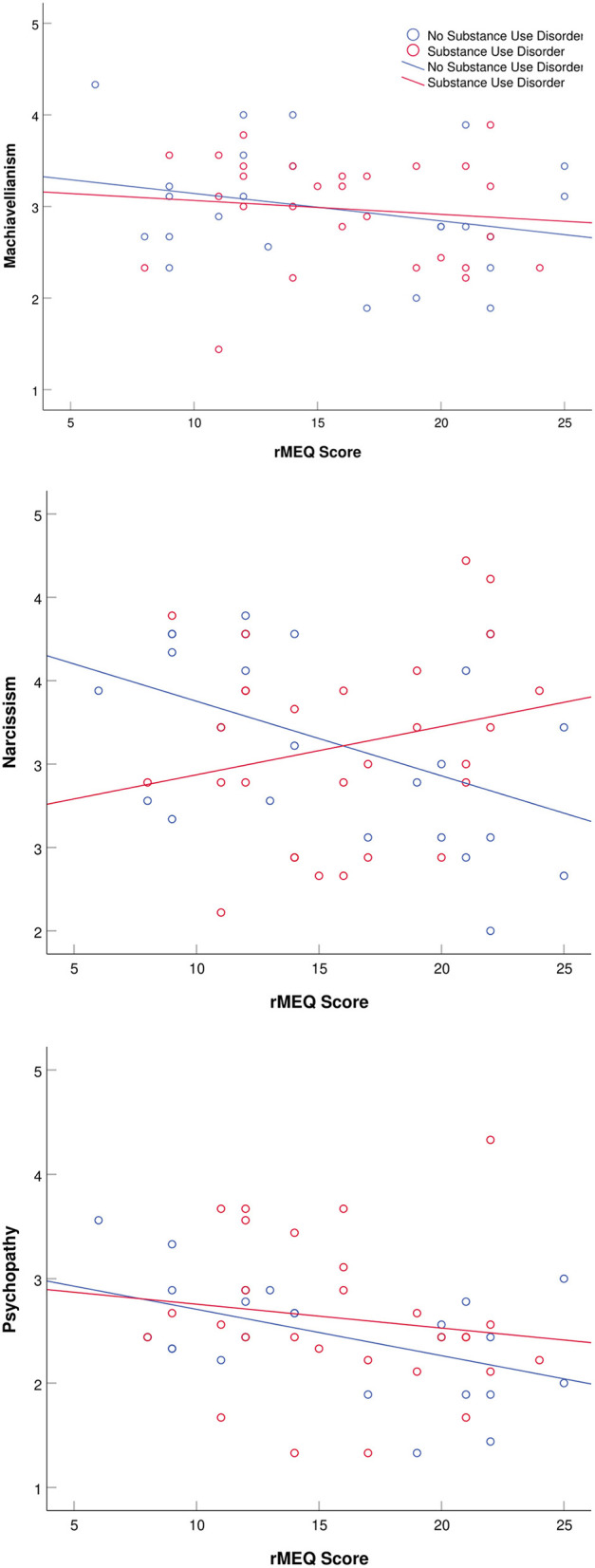
Scatter plots dark triad trait scores and reduced Morningness–Eveningness Questionnaire (rMEQ) scores separated by substance use disorder and non-substance use disorder populations. Low rMEQ scores indicate preference eveningness and high scores indicate preference for morningness.

Subjects not diagnosed with a psychotic disorder tended to score higher on anger and hostility as eveningness increased (Figure 1). Substance use disorder subjects were generally more narcissistic among morning chronotypes, whereas eveningness was associated with greater narcissism when a substance use disorder was not present (Figure 2). None of these relationships was statistically significant.

## Discussion

To our knowledge, this study is the first to evaluate chronotype in forensic psychiatric inpatients. Our analyses were primarily exploratory and are thus most relevant to aggressive, institutionalized males. We report that 25% of the forensic patients sampled were classified as being evening types, and 36% as morning types. This is in contrast to healthy cohorts, that have identified up to 15% and 54% as being evening and morning types, respectively (4, 6, 13). Patients diagnosed with a personality disorder had a significantly greater preference for eveningness. Of the 22 subjects with a personality disorder, 13 of these individuals had a comorbid psychotic diagnosis. Positive associations between eveningness and anger, as well as eveningness and hostility were found in forensic patients without psychosis. In substance use disorder patients, we observed that greater narcissism scores were associated with morningness.

Findings of the present study showed increased eveningness in those diagnosed with a personality disorder. This was not reflected in MSF_SC_ and social jetlag, indicating that chronotypic differences between these two groups are perhaps more related to individual preference for wake and sleep, and less so to actual sleep timing. Despite the stark contrast in anti-psychotic medication prescribed to patients with psychotic disorders vs. those without, mean anti-psychotic dose did not correlate with chronotype (raw scores or type) and thus it seems unlikely that increased eveningness in personality disorders may be attributed solely to medication effects. Personality disorder patients in this study spent less time in bed and slept fewer hours each night, when compared to the non-personality disorder population; however, the contribution was not substantial enough to impact MSF_SC_. It is possible that significant deviations in sleep times were mitigated by the daily structure provided by the hospital environment. Similarly, an epidemiological survey of three inpatient psychiatric clinics found that patients with a personality disorder were more likely to be the evening type (8). One study reported increased morningness for patients with ASPD comorbid with substance use disorder (10); however, participants were outpatients of an addictions clinic seeking a treatment specifically for substance use. In our forensic sample, ASPD was the most common personality disorder among participants (data not shown). Antisocial personality traits that are most frequently associated with the evening chronotype include extreme irresponsibility, disregard for societal norms, and impulsivity that often manifests as the inability to plan ahead (12, 18). Interestingly, a research study on sleep among habitually violent offenders with ASPD determined that forensic psychiatric patients experienced greater slow-wave sleep than healthy controls (36). While these findings do not directly implicate chronicity, they support the notion that diagnosis may be linked to identifiable differences in sleep.

Psychosis patients in this study spent more time in bed, slept longer, had a higher mean anti-psychotic dose, but did not differ in chronicity (rMEQ, MSF_SC_, or social jetlag) from the non-psychosis population. Anti-psychotic mean dose may have lessened the degree of eveningness observed in the psychosis population as drowsiness is a common side effect of psychotropic medication (37). Two notable studies on psychosis and chronotype report morningness in an inpatient population (8) and eveningness in outpatients (7). Outpatient and community populations have been shown to have higher rates of medication non-compliance (38, 39) and thus we suggest that chronotype in psychosis patients may be influenced by anti-psychotic medication treatment.

In our sample, forensic patients without psychosis showed a positive association between eveningness and anger, as well as eveningness and hostility. The association of anger and impulsivity with eveningness has similarly been reported in community populations (13). Deibel et al. (40) posited that mild yet chronic circadian misalignment associated with an evening type may contribute to the expression of aggressive behavior. Social jetlag is a mild yet chronic type of circadian misalignment experienced by a surprisingly large portion of the population (41–44). Social jetlag involves adhering to a schedule that is in conflict with one's natural circadian phenotype [for review see (44)]. For example, this can be evidenced by someone with an evening preference accumulating a sleep debt during the work week that is alleviated on the weekend by sleeping in, thus making it harder to entrain come Monday and creating a state of chronic circadian misalignment (42). We failed, however, to find that social jetlag was associated with diagnosis, aggression, or dark triad traits. Few studies assessing aggression have measured social jetlag. Vollmer and Randler (45) did find that social jetlag positively correlated with physical aggression. The disparity with the current report could be due to our patients having more control in their weekday schedule than those who are working or in school. This means that in our sample a “weekday” might be more similar to a “weekend” than in a non-institutionalized setting, thus negating the impact of social jetlag.

Patients in this study reported generally poor quality of sleep (e.g., greater PSQI scores), with eveningness being associated with poorer sleep quality. Although we were unable to identify a significant relationship in our modest sample, self-reported aggression has been found to be associated with poor sleep quality, rather than simply being part of general psychopathology (46). In terms of poor sleep quality, our findings are consistent with the literature among inpatients (47), where psychiatric patients have been identified as being affected to a greater degree when compared with non-psychiatric hospitalizations (48).

Forensic patients diagnosed with a substance use disorder showed a positive association between chronotype (e.g., morningness) and narcissism scores, whereas the non-substance use disorder population had a negative association between chronotype (e.g., eveningness) and narcissism. These findings are discordant with the literature as substance abuse has been previously linked to eveningness (10) and to narcissism (49). However, participants diagnosed with a substance use disorder in this study had at least one other comorbid condition (e.g., schizophrenia or personality disorder), highlighting the complexity of forensic psychiatric populations. It is also worth noting that all participants in our study were institutionalized and unable to access drugs or alcohol. Thus, a sustained period of sobriety may have influenced chronicity when compared to actively using individuals. These findings shed light on a potential mediating effect that substance use may exert on chronotype when it is comorbid with a psychiatric condition.

The present study has several limitations. Our findings are based on a relatively small, primarily male population, and we did not sample a healthy control group. Participant recruitment for this study required that subjects not have any major mental impairments that would affect their ability to provide informed consent and thus we can only infer how these results may compare to the forensic population as a whole; this is a common limitation of studies that evaluate populations with severe mental illnesses. The presence of comorbidities limited our ability to parse out the effects of an individual disorder. Although this issue could be ameliorated by identifying the mental illness as being primary or secondary, the diagnoses were determined by clinicians, and they did not designate diagnoses as primary or secondary. Our data are cross-sectional; thus, we cannot infer causality between chronotype, mental disorder, aggression, and dark triad traits.

In conclusion, we investigated chronotype, aggression, and dark triad traits in a forensic psychiatric inpatient population. We report that one quarter of the participants were classified as evening types and approximately one third were morning types, which differs from the chronotypes observed in the general population (13). Participants diagnosed with a personality disorder were more likely to be evening types. Increased eveningness was reflected only in general timing preferences, but not in sleep timing, perhaps indicating the moderating role of a highly structured, institutionalized environment. This association may be specific to ASPD, as it was the most common personality disorder in our study. Patients with psychosis showed no preference for eveningness or morningness, a trend we attribute to anti-psychotic medication. In the non-psychosis population, there was an association of eveningness with anger and hostility. Furthermore, poor sleep and increased evening types are consistent with what has been associated with aggressive populations. Patients with a substance use disorder showed an association of morningness and increased narcissism; however, narcissism scores in patients not diagnosed with a substance use disorder were associated with eveningness. The sample size prevented any determination based on substance preference or the relative contribution of sobriety on chronotype. Given the unique sample composition, these findings are most relevant to forensic psychiatric males. Future research should evaluate the role of comorbidity in determining chronotype and give special attention to specific personality disorder and substance use disorder subtypes.

## Data Availability Statement

The raw data supporting the conclusions of this article will be made available by the authors, without undue reservation.

## Ethics Statement

This study involving human participants was reviewed and approved by The Research Ethics Board at Waypoint Centre for Mental Health Care, Penetanguishene, Ontario, Canada. The patients/participants provided their written informed consent to participate in this study.

## Author Contributions

Study design and conception: SD and NK. Statistical analysis: KB. Writing and review of manuscript: KB, SD, and NK. All authors contributed to the article and approved the submitted version.

## Conflict of Interest

The authors declare that the research was conducted in the absence of any commercial or financial relationships that could be construed as a potential conflict of interest.
